# Left Upper Extremity Pain, Right Coronary Artery Culprit: A Puzzling Path to Aneurysm Discovery

**DOI:** 10.14797/mdcvj.1287

**Published:** 2024-04-05

**Authors:** Moath Said Alfawara, Vivek Modi, Min-Fang Chao, Malek Nayfeh, Fares Alahdab, Mahmoud Alrifai, Mouaz Al-Mallah

**Affiliations:** 1 Houston Methodist DeBakey Heart & Vascular Center, Houston Methodist, Houston, Texas, US

**Keywords:** giant coronary artery aneurysm, atherosclerosis, right coronary artery, multimodality cardiac imaging

## Abstract

Giant coronary artery aneurysm (GCA) is a rare disease afflicting 0.2% of the population. It is primarily attributed to atherosclerosis in adults and Kawasaki disease in children. Other uncommon etiologies include Takayasu arteritis and post-percutaneous coronary intervention.^1,2^ GCA lacks a universally accepted definition, with proposed criteria including a diameter exceeding 2 cm, 5 cm, or four times the normal vessel size.^3^ While the majority of GCAs are asymptomatic, a subset of patients present with angina, myocardial infarction from embolization or compression, heart failure due to fistula formation, or even sudden death.^1^ We report a case of an adult harboring a GCA involving the right coronary artery.

## Case Presentation

A 71-year-old male with a medical history of hypertension, hyperlipidemia, and obstructive sleep apnea presented to the emergency department (ED) on multiple occasions with left upper extremity pain. The patient has a family history of premature coronary artery disease, with his father having undergone coronary artery bypass grafting in his 50s. The patient’s pain was initially persistent but later became intermittent. He described it as a pressure-like sensation radiating to the lower left arm, exacerbated by exertion and alleviated by rest. The pain was not accompanied by any other cardiac or respiratory symptoms. Electrocardiogram did not show any ischemic changes. Serial high-sensitivity troponins remained normal. Echocardiography revealed a hypokinetic inferior wall with a rounded structure adjacent to and slightly compressing the right atrium (Video 1).

**Video 1:** Subcostal echocardiography view showing a rounded structure measuring 6.8 cm in diameter adjacent to and slightly compressing the right atrium; see also at https://youtube.com/shorts/k97OoTMpAbo.

**Figure 1:**
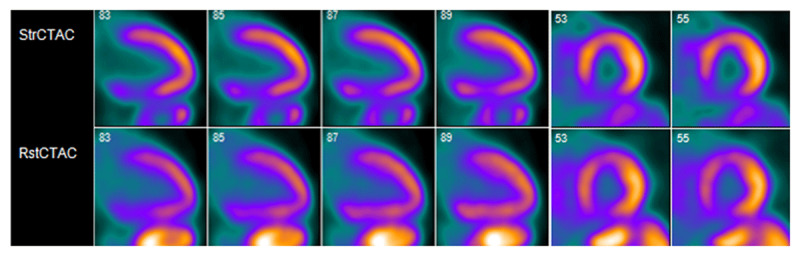
Cardiac positron emission tomography showing a medium-sized, moderate-severity, partially reversible defect in the proximal and mid inferior and inferoseptal segments.

Subsequently, positron emission tomography pharmacological stress testing demonstrated an infarct with peri-infarct ischemia in basal to mid inferior and inferoseptal walls (Figure 1). The patient underwent cardiac catheterization, which revealed an aneurysmal left coronary system. There was an unusual posterior takeoff of an ectatic right coronary artery (RCA) that could not be completely opacified (Video 2).

**Video 2:** Coronary angiogram showing an ectatic left coronary system and an incompletely opacified ectatic proximal right coronary artery; see also at https://youtube.com/shorts/0ssjghqx5sM.

**Figure 2:**
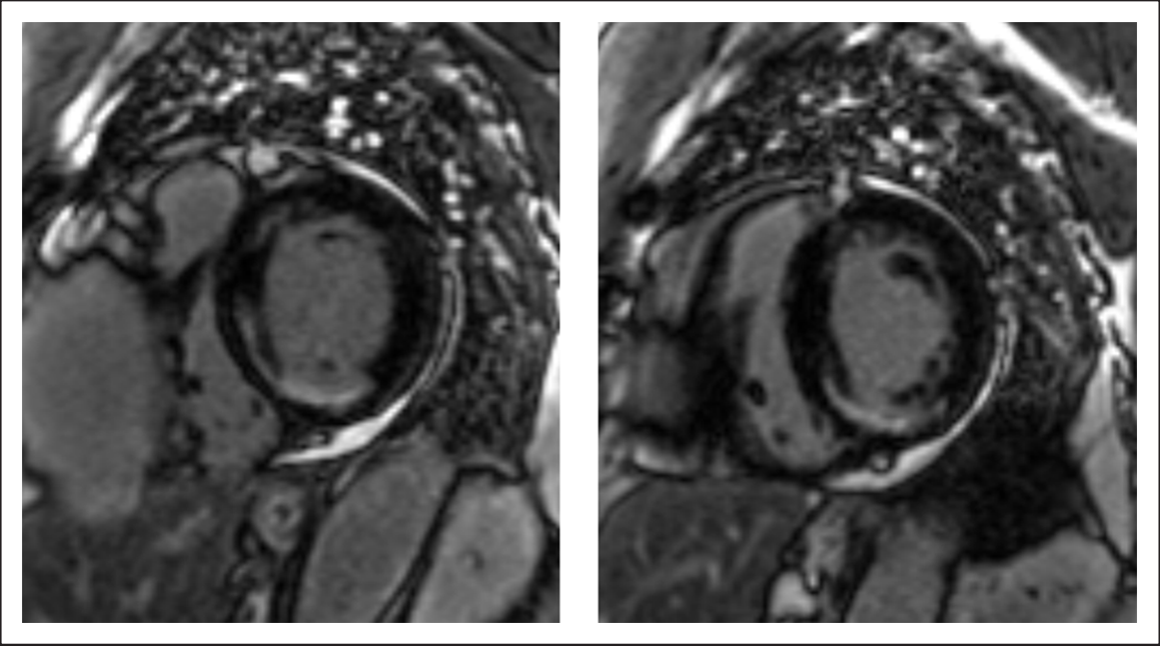
Late gadolinium enhancement cardiac magnetic resonance imaging showing left ventricular subendocardial scar in the basal and mid inferior and inferoseptal walls consistent with right coronary artery related myocardial infarction.

Cardiac computed tomography revealed a giant aneurysm of the RCA measuring 9 × 11 cm that originated from the posterior aspect of the right-coronary cusp closer to the non-coronary cusp. The proximal RCA was patent, with a small jet of contrast extending into the cephalad portion of the aneurysm sac posteriorly. The aneurysm was compressing the right atrium (Video 3). Cardiac magnetic resonance imaging confirmed an aneurysmal RCA and revealed subendocardial late gadolinium enhancement in the basal and mid-inferior segments, indicating partial viability of RCA territory (Figure 2).

**Video 3:** Cardiac computed tomography angiography sagittal view showing a giant retrosternal right coronary artery aneurysm extending from the proximal to distal segment, compressing the right atrium; see also at https://youtu.be/HMw3lXG_90c.

Due to recurrent anginal symptoms requiring multiple ED visits, a multidisciplinary decision was made for resection of the aneurysm and bypass of RCA. Intraoperatively, an ectatic proximal RCA was seen feeding into the aneurysmal sac, and a rather small RCA was seen exiting the aneurysmal sac. The patient underwent surgical excision of the aneurysm, and the distal RCA was dissected and ligated. The posterior descending artery was opened and grafted end-to-side with a saphenous vein. The resected aneurysm was sent for pathology, which revealed a myxoid degeneration of the media. The patient successfully underwent cardiac rehabilitation with no recurrence of anginal symptoms at the 6-month follow-up.

## Discussion

While giant coronary artery aneurysms (GCAs) can manifest in any coronary artery, a propensity for the RCA, especially proximally, has been documented.^4,5^ Due to the rarity of GCAs, there are no large, randomized studies to guide management decisions. Surgical techniques such as ligation with bypass, isolated bypass, and plication are preferred for large aneurysms that present with mass effect or pose a high risk of rupture or distal thromboembolism.^1,3^ Successful short-term conservative management with warfarin and antianginals has been reported in asymptomatic patients.^6^
